# Illuminating traffic control for cell–division planes

**DOI:** 10.7554/eLife.02747

**Published:** 2014-04-08

**Authors:** Silke Robatzek

**Affiliations:** 1 **Silke Robatzek** is at The Sainsbury Laboratory, Norwich, United Kingdomrobatzek@TSL.ac.uk

**Keywords:** post-Golgi trafficking, regulation of vesicle traffic, ARF-GEF, cell division, secretion, recycling, endocytosis, Arabidopsis

## Abstract

When a plant cell divides, four related proteins control the trafficking of vesicles and ensure that cargo that is normally recycled to the plasma membrane is instead re-routed to the plane of cell division.

**Related research article** Richter S, Kientz M, Brumm S, Nielsen ME, Park M, Gavidia R, Krause C, Voss U, Beckmann H, Mayer U, Stierhof Y-D, Jürgens G. 2014. Delivery of endocytosed proteins to the cell–division plane requires change of pathway from recycling to secretion. *eLife* **3**:e02131. doi: 10.7554/eLife.02131

**Image** When a plant cell divides, a ‘cell plate’ (stained in green) forms at the plane of cell division

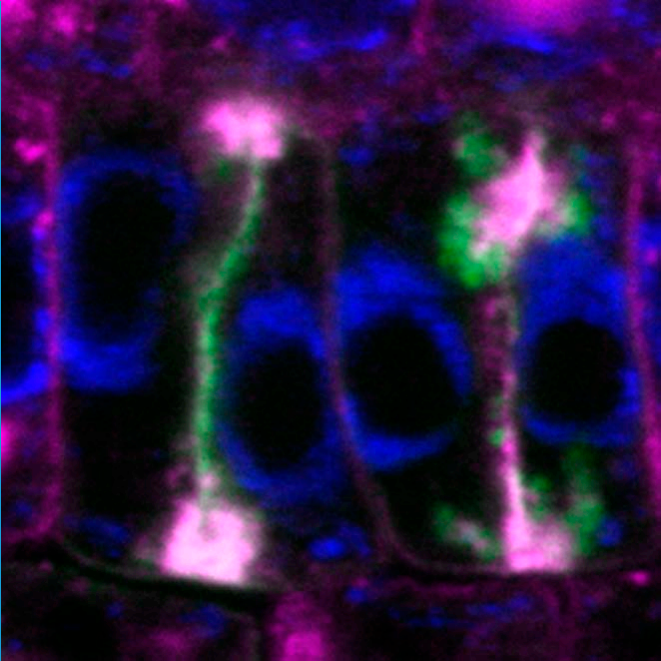

In 1875, the German botanist, Eduard Strasburger—who is today acknowledged as one of the fathers of modern cell biology—uncovered the process by which a single cell divides into two daughter cells (Volkmann et al., 2012). Under a microscope, Strasburger saw bubble-like structures that moved to the centre of the dividing plant cell, and then fused to produce a ‘cell plate’ that eventually separated the cell into two. Today, almost 140 years later, we have learned much about how the cell plate is formed, but many of the details of how this process is controlled remain poorly understood. Now, in *eLife*, Gerd Jürgens of the University of Tübingen and co-workers—including Sandra Richter as first author—have identified an important transition that underpins the movement of these bubble-like structures, called vesicles, to the cell plate (Richter et al., 2014).

Vesicles are membrane-bound packages that transport molecules within a cell: for example, they transport proteins from the endoplasmic reticulum (where they are synthesised and folded) to the Golgi (where they are sorted and packaged for transport to other destinations). Vesicles also deliver proteins to be inserted into the plasma membrane or secreted to the outside of the cell; and they remove material from the plasma membrane or cell exterior (via endocytosis) so that it can be recycled or degraded.

Eukaryotic cell division, or cytokinesis, occurs in different ways in animals and plants. Animal cells contract their plasma membrane inwards from the cell periphery, and fill in the gap with material from vesicles (Schiel et al., 2013). By contrast, plant cells divide by forming a new membrane within the cell—the cell plate—that grows outwards until it joins with the existing plasma membrane. Vesicles are directed to the cell plate by the ‘phragmoplast’: this is a scaffold-like structure that forms within a plant cell before the cell divides.

Thanks to advances in microscopy and the wealth of knowledge gained from studying the model plant *Arabidopsis*, it is now well established that the phragmoplast delivers vesicles from the *trans*-Golgi network to the cell plate (Jürgens, 2005). The fusion of these vesicles is mediated by protein complexes called SNAREs (El Kasmi et al., 2013), whilst clathrin-coated vesicles mediate the endocytosis of excess membrane material away from the cell plate (Jürgens, 2005). More vesicles fuse to the margins of the cell plate, expanding it until it eventually merges with the existing plasma membrane to complete cell separation.

In plants, the *trans*-Golgi network acts as a hub for three different trafficking routes—it sorts the cargo that comes from the Golgi for secretion and is also involved in recycling and endocytic trafficking (Viotti et al., 2010). This raises interesting questions about how cargo transported via vesicle trafficking is directed to the appropriate destination. Earlier seminal studies revealed that the protein GNOM regulates the recycling of material from, and back to, the plasma membrane (Geldner et al., 2003). GNOM belongs to a family of eight proteins in *Arabidopsis* that together are called ‘ARF-GEFs’: this family also include two GNOM-like proteins and five members of the BIG subfamily. GNOM-like 1 controls the transport of material from the Golgi back to the endoplasmic reticulum (Teh and Moore, 2007; Richter et al., 2011), while BIG5 is involved in endocytic trafficking (Tanaka et al., 2009).

Jürgens and co-workers—who were also based at the University of Tübingen—combined genetic, chemical and imaging approaches to decipher the functions of BIG1 to BIG4 (BIG1-4; Richter et al., 2014). This revealed that these proteins normally control secretory (and late endosomal) cargo trafficking, and are not involved in endocytic recycling. However, they undergo an important transition during cell division to redirect vesicles from recycling to secretory trafficking (Figure 1).

**Figure 1.**
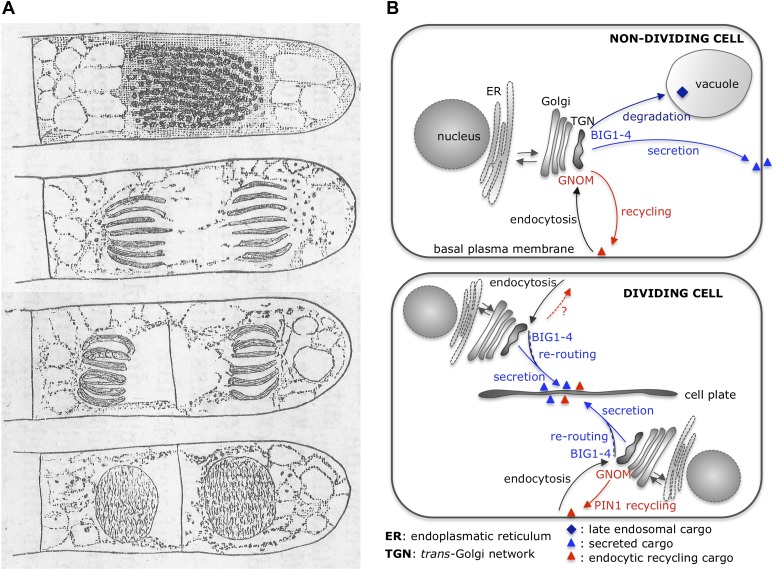
Cytokinesis: past and present. (**A**) Eduard Strasburger’s drawings (from 1884) of a plant cell at different stages of division show a cell plate forming. Drawings are courtesy of Dieter Volkmann, and the complete figure can be seen in Volkmann et al., 2012. (**B**) After arrival at the *trans*-Golgi network (TGN), newly synthesized proteins from the Golgi or previously endocytosed cargo proteins are sorted or recycled back to the plasma membrane, respectively, or trafficked to the vacuole (via late endosomal trafficking). Richter et al. have shown that, in non-dividing cells, four proteins (BIG1-4) belonging to the ARF-GEF family control secretory trafficking and transport to the vacuole. Another ARF-GEF, GNOM, regulates endocytic recycling of proteins, such as PIN1. In dividing cells, a plant-specific transient membrane compartment, the cell plate, is formed via BIG1-4-mediated trafficking of vesicles along the secretory pathway, but also via BIG1-4-mediated re-routing of endocytic recycling cargo to the cell-division plane. Interestingly, GNOM-dependent polar relocalisation of PIN1 is maintained despite BIG1-4 re-routing the endocytic recycling cargo.

Illustrations by Eduard Strasburger.

Studies on the role of ARF-GEFs have to overcome the redundancy within this protein family. However, some of the ARF-GEFs (including GNOM) are sensitive to the fungal toxin Brefeldin A; shorted to BFA (Geldner et al., 2003). This toxin provides a useful tool to inhibit these proteins and block trafficking to the plasma membrane (Langhans et al., 2011), while the production of BFA-resistant and sensitive versions of ARF-GEFs expands this tool’s usefulness (Geldner et al., 2003; Teh and Moore, 2007). Efforts to engineer plants in which all four *big1-4* genes had been knocked-out were hampered by defects in pollen growth: however, Richter et al. revealed that BIG1, 2, and 4 are inhibited by BFA, while BIG3 is resistant to this toxin. Hence, applying BFA to plants with only the *big3* gene knocked-out actually inhibited the redundant function of all four proteins—and using a BFA-resistant GNOM confirmed which effects were specifically due to inhibiting BIG1-4.

The proteins BIG1-4 control the trafficking of cargo from the *trans*-Golgi network to the plasma membrane, and also to the vacuole—a large membrane-enclosed compartment within a plant cell (Richter et al. 2014). BIG1-4 deliver newly synthesized proteins, including the auxin efflux carrier PIN1, to the plasma membrane. Recycling of pre-existing molecules of PIN1—from and to the plasma membrane—is controlled instead by GNOM (Geldner et al., 2003), and relocates the efflux carrier to one side of a root cell. This relocalisation allows roots to grow downward in response to gravity, which means that these observations are in agreement with the fact that, unlike GNOM, BIG1-4 are not involved in the root ‘gravitropic response’ (Richter et al., 2014).

Richter et al. also discovered that, when a plant cell is dividing, the BIG1-4 proteins do more than transport secretory cargo to the cell plate—they also redirect endocytic cargoes from the recycling to the secretory pathway towards the cell–division plane (Richter et al., 2014). Again, these observations are consistent with cell plate defects (and binucleated cells) that occur when BIG1-4 function is inhibited. Interestingly, endocytic PIN1 appears a special cargo, as here GNOM works against BIG1-4 to maintain its polar plasma membrane localization even during cell division. Richter et al. discuss that this antagonism could allow PIN1-mediated auxin fluxes to be maintained during cell division, which is particularly important in processes such as lateral root formation.

More needs to be learned about how BIG1-4 and GNOM regulate different aspects of vesicle cargo delivery: Are they located at distinct domains within the same compartment, or are they present at different *trans*-Golgi network compartments (as previously discussed by Chow et al., 2008)? Detailed co-localization studies in dividing cells and non-dividing cells over time will help address these questions. What is the molecular mechanism behind the GNOM-BIG1-4 antagonism, and how does GNOM have priority over BIG1-4 in the case of PIN1 recycling? Is PIN1 the only exceptional cargo or does the antagonism between GNOM and BIG1-4 have different outcomes for different groups of proteins? And last, but not least, we still do not how ARF-GEFs determine the trafficking route that a given cargo should take and, in particular, whether or not this involves post-translational modifications of the cargo itself.
